# Effects of increasing lipopolysaccharide concentrations on *in vitro* developmental competence of ovine oocytes

**DOI:** 10.1590/1984-3143-AR2019-0125

**Published:** 2020-07-07

**Authors:** Sepideh Heydari, Akram Eidi, Fatemeh Kouhkan, Eva Tvrda, Abdollah Mohammadi-Sangcheshmeh

**Affiliations:** 1 Department of Biology, Science and Research Branch, Islamic Azad University, Tehran, Iran; 2 Department of Molecular Biology and Genetic Engineering, Stem Cell Technology Research Center, Tehran, Iran; 3 Department of Animal Physiology, Faculty of Biotechnology and Food Sciences, Slovak University of Agriculture in Nitra, Nitra, Slovakia; 4 Department of Animal and Poultry Science, College of Aburaihan, University of Tehran, Pakdasht, Tehran, Iran

**Keywords:** embryo, GSH, *in vitro* fertilization, sheep oocyte

## Abstract

Although a considerable number of studies have investigated the effects of lipopolysaccharide (LPS) on the reproductive performance of dairy cows, the response of ovine oocytes to LPS during their *in vitro* maturation and development is not well defined yet. Ewe’s ovaries were obtained from a slaughterhouse, the oocytes were collected and matured in the presence of increasing concentrations (0, 0.01, 0.1, 1 and 10 µg/mL) of LPS in order to evaluate the meiotic maturation by measuring the proportion of oocytes reaching the MII stage. The concentration of intracellular glutathione (GSH) was measured in oocytes following maturation *in vitro*. In addition, concentrations of selected metabolites including glucose, pyruvate, lactate and glutamine were quantified in the medium following maturation. A number of treated matured oocytes along with the control group were subsequently fertilized using frozen semen and assessed for the rate of cleavage and for the proportion reaching the blastocyst stage. The number of oocytes in MII stage was significantly reduced in response to the increasing concentrations of LPS (77.83%, 70.64%, 68.86%, 66.32%, respectively, in case of 0.01, 0.1, 1 and 10 µg/mL LPS when compared to the control group, 76.34%; *P<0.05*). There were no differences neither in the intracellular concentration of GSH in the oocytes nor in case of the metabolites in the maturation medium. Although the rate of cleaved oocytes was not affected by increasing levels of LPS, the blastocyst rate was reduced in a dose dependent manner (36.69%, 34.21%, 30.35%, 17.27% and 14.03% for the control, 0.01, 0.1, 1 and 10 µg/mL LPS, respectively (*P<0.05*). These results demonstrate that the developmental competence of ovine oocytes may be affected detrimentally by LPS and such deleterious effects could be related to the maturation process.

## Introduction

Postpartum uterine infections in ewes have not been studied as much as the respective issue in cows. Although these happen less often than in cows, they can yet be considered as a cause of environmental death in sheep (Ioannidi et al., 2020). Infectious diseases enhance the concentration of lipopolysaccharide (LPS), an important bacterial component in the circulation (Dong et al., 2011). In recent years, the role of LPS got much attention due to its ability to be transmitted from the gastrointestinal tract, mammary glands, and uterus to the bloodstream (Eckel and Ametaj, 2016). LPS is a glycolipid found in the outer membrane of the cell wall of gram-negative bacteria (Mani et al., 2012). Toll-like receptors (*TLRs*) are one of the pathogen pattern recognition receptor families that can identify LPS (Schumann et al., 1990). When LPS reacts with *TLR*, it triggers an intracellular signaling cascade that causes the release of pro-inflammatory cytokines such as *IL-1*, *TNFα*, and toxic free radicals (Murphy et al., 2010). *TLRs* signaling has been subdivided into *MyD88*-dependent and *MyD88*-independent (*TRIF*-dependent) pathways. *Myd88*-dependent signaling leads to the activation of nuclear *factor-kappaB* (*NF-κB*) and mitogen activated protein kinase (*MAPK;* specifically *p38*, *JNK*), which can cause the expression of early response genes including pro-inflammatory cytokines (Mogensen, 2009; Newton and Dixit, 2012; Takeda and Akira, 2015). On the other hand, the independent *MyD88* pathway culminates in the activation of the *TRIF* adapter, which leads to the expression of *IFN-β* and interferon genes (Tserel et al., 2011). The *MAPK* cascades are involved in regulating the expression of many genes in various cellular processes including cell growth, differentiation, and apoptosis (Plotnikov et al., 2011). Cumulus-oocyte complexes (COCs) exposed to LPS enhance the level of *p38 MAPK* phosphorylation that causes increased expression of *NF-κB* and *IL-6* (Shimada et al., 2006). LPS was found in the follicular fluid of cows diagnosed with endometritis and/or mastitis demonstrating a relationship between uterine infection, LPS production and follicular function (Herath et al., 2007; Magata et al., 2015). It has been reported that granulosa cells can initiate an inflammatory response about the pathogen associated pattern (*PAMPs*; Bromfield and Sheldon, 2011). The concentration of 10 µg/mL LPS prevented bovine oocyte maturation by affecting the cell cycle, oxidative stress and epigenetic alterations resulting in a reduced polar body extrusion rate (Zhao et al., 2017). In addition, it reduced the rate of cleavage, morula and blastocyst formation in bovine oocytes (Zhao et al., 2019). Many studies have shown that LPS in various cell lines including macrophages, epithelial and dendritic cells can decrease the cytotoxicity by increasing the cellular oxidative stress as LPS exposure could alter the intracellular glutathione-dependent redox homeostasis (Zhao et al., 2017). It has been postulated that the effects of LPS on oocyte developmental competence depends on the activation of antioxidant systems. Although several studies investigated the effects of LPS on the reproductive performance of dairy cows (Bromfield and Sheldon, 2013; Magata et al., 2014; Zhao et al., 2019), the response of ovine oocytes to increasing concentrations of LPS is not well defined yet. Ewes do not experience negative energy balance (NEB) and metabolic stress after parturition as much as dairy cows, however severe feed shortage and high numbers of suckling lambs can predispose milking ewes to NEB, weaken the immune system and consequently their susceptibility towards infectious diseases such as metritis and mastitis. Different energy substrates in the maturation medium can affect the nuclear and cytoplasmic maturation of the oocyte (Downs and Hudson, 2000). Embryogenesis is strongly affected by the conditions of oocyte maturation (Sutton-McDowall et al., 2004). The cumulus cells around the oocyte communicate with the oocyte through gap junctions (Allworth and Albertini, 1993), which allow the transfer of metabolites (Gilula et al., 1978). Glucose, pyruvate, and lactate are major substrates for energy metabolism in the cumulus cells (Rieger and Loskutoff, 1994). Oocytes have a limited ability to use glucose but the molecule can be metabolized in the cumulus cells via glycolysis to form pyruvate or lactate which can be subsequently utilized by oocytes (Sutton-McDowall et al., 2004). Pyruvate is the principal energy substrate, which can be used directly by the oocytes and zygotes (Gwatkin and Haidri, 1973). Maturation of the COCs in glucose-free medium leads to aberrations in the expansion of the cumulus cells and embryo development (Rose-Hellekant et al., 1998). Moreover, glutamine is one of the main substrates for the synthesis of hyaluronic acid, so its deficiency in the culture medium leads to a disruption of nuclear maturation (Hashimoto et al., 2000). Accordingly, metabolites such as glucose, lactate, pyruvate and glutamine are intermediate substrates of the cell, and their concentrations in the maturation medium can be used to evaluate the cellular and tissue metabolism. To our knowledge, this study is the first report of analyzing the glucose, lactate, pyruvate and glutamine levels in the maturation medium following the LPS challenge. Hence, this study aimed initially to determine the effects of LPS in the maturation and culture medium on the ovine oocyte developmental competence. Secondly, we aimed to elucidate the suitability of metabolites from the maturation medium to further understand and/or predict the LPS induced inflammation mechanisms.

## Methods

### Ethics statement

The current study was performed according to the procedures established by the Iranian Ministry of Agriculture (experimental permission 858). All chemicals and reagents used in this study were purchased from Sigma-Aldrich Chemical Company (St. Louis, MO, USA) and Gibco (Grand Island, NY, USA), unless otherwise stated.

### Ovary collection

Immediately after slaughtering, ovaries were obtained from ewes at a local slaughterhouse (Varamin, Tehran, Iran) and transported within 1-2 h in thermo flask containing 0.9% saline (32-37 °C) to the laboratory.

### Aspiration of follicular fluid

COCs were collected from the antral follicles using the aspiration method. Follicular fluid was aspirated using a 5 mL syringe (18 G needle) and searched for the COCs under the stereomicroscope. High quality oocytes, which were completely surrounded by a compact and thick cumulus with 2 or 3 layers and with a homogeneous cytoplasm were selected for the subsequent experiments.

### *In vitro* Maturation (IVM)

Selected COCs were washed three times in a maturation medium consisting of bicarbonate-buffered TCM-199 with 2 mM glutamine supplemented with 10% fetal bovine serum, 5.5 mg/mL sodium pyruvate, 25 mg/mL gentamycin sulphate, 5.0 μg/mL LH, 0.5 μg/mL FSH, and 1 μg/mL estradiol. A group of 10 COCs were cultured in a 50 μL drop of maturation medium in a 30 mm petri dish for 24 h at 38.5 °C in 5% CO_2_ of maximum humidified air.

### Oocyte nucleus evaluation

After 24 h, the cumulus cells were removed from oocytes mechanically using hyaluronidase (600 IU/mL) in TCM-199 and the oocytes were subsequently stained with 2.5 mg/mL Hoechst 33258 in 3:1 (v/v) glycerol/PBS. Oocytes from each group were evaluated for the first polar body extrusion using an epifluorescence microscope (Nikon Eclipse-600). The presence of second metaphase plate and first polar body or two independent areas of fluorescing chromatin in the oocytes were classified as Metaphase II (MII; Mohammadi-Sangcheshmeh et al., 2011, 2012).

### Measurement of intracellular glutathione (GSH)

Immediately after IVM, COCs were denuded and incubated in Tyrode’s medium plus 5 mg/mL polyvinyl alcohol containing 10 μM Cell Tracker Blue for 30 min. The oocytes were subsequently washed in PBS, placed into 10 μL droplets, observed under an epifluorescence microscope (Nikon, Tokyo, Japan) with UV filters, and all fluorescent images were recorded as graphic files. The images taken from oocytes were then analyzed by ImageJ software program (Abràmoff et al., 2004).

### Measurements of metabolites in maturation medium following IVM

Following IVM, the maturation medium was collected and in order to remove all impurities, the cumulus cell suspension was centrifuged at 5000 rpm for 15 min at 4 °C and the supernatant was discarded. Then medium was kept at -20 °C until to be analyzed for glucose, pyruvate, lactate and glutamine. Glucose and lactate measurement were based on a photometric assay using commercial diagnostic kits (Pars Azmoon Inc., Tehran, Iran). Pyruvate was measured with an enzymatic method and GRINER kit (7031500). Glutamine was determined using high performance liquid chromatography (HPLC).

### *In vitro* Fertilization (IVF)

Following IVM, COCs were washed twice in HSOF and once in IVF medium [SOF supplemented with 4 IU/mL heparin, PHE (20 mM penicillamine, 10 mM hypotaurine, 1 mM epinephrine), and 2% (v/v) sheep serum], subsequently placed in 50 μL drops of IVF medium covered with mineral oil. Frozen semen from a ram was used for IVF. A single straw of semen was thawed at 37 °C for 30 sec, spermatozoa were centrifuged at 500 g for 10 min, washed twice with Sperm Tyrode’s Albumin Lactate Pyruvate medium (Sperm-TALP) containing 10 μg/mL heparin, 2.2 mg/mL sodium pyruvate and bovine serum albumin (BSA) F-V (6 mg/mL) + 50 μg/mL gentamycin. After washing, the sperm pellet was suspended in 0.5 mL of fresh Fert-TALP medium supplemented with 6 mg/mL BSA (fatty acid free) + 10 μg/mL heparin + 3 μL PHE and 50 μg/mL gentamycin. Sperm concentration was adjusted to 2 × 10^6^ spermatozoa/mL. The washed and suspended sperm cells were incubated with mature COCs for 18 h under 5% CO_2_ in humidified air at 39 °C (Mohammadi-Sangcheshmeh et al., 2012).

### *In vitro* Culture (IVC)

Following IVF and after 18 h, the presumable zygotes were denuded and washed three times in CR1aa medium. Groups of 15 to 20 zygotes were cultured in a 30 μL CR1aa (2% BME essential amino acids, 1% MEM-nonessential amino acids) medium drops supplemented with 10% FBS for 9 days at 38.5 °C in a humidified incubator with 5% O_2_, 5% CO_2_, and 90% N_2_. The day of fertilization was considered as day 0. The stage of embryonic development was evaluated at day 3 and 9 post-fertilization.

### Statistical analysis

Logistic regression analysis was performed to determine the association between the dependent variable (oocytes nuclear maturation, cleavage and blastocyst rates) and independent variables (experimental treatments) using GLM (generalized linear model) function with binomial family and logit link in the R (3/1/0) statistical software. The strength of the association was estimated by an odds ratio (OR) measure. The intracellular GSH level and metabolites content in maturation medium were analyzed based on a completely randomized design using GLM procedure of SAS software package version 8.0 (SAS Institute Inc., NC, USA) and orthogonal comparison was used to test the increasing level of LPS with the level of significance of *P<0.05*.

### Experimental design

#### Experiment 1. Effects of LPS in maturation medium on the nuclear status and GSH content of oocytes

In this experiment, different concentrations (0, 0.01, 0.1, 1, and 10 μg/mL) of LPS were applied during oocyte maturation *in vitro*. After IVM, COCs were evaluated for their nucleus phase. Ten replicates were performed per treatment.

The oocytes were then analyzed for the GSH content. For this, image of each oocyte was recorded and analyzed with Image J software (ImageJ, 2019) to quantify objectively the amount of GSH. Five replicates were performed per treatment.

#### Experiment 2. Effects of LPS in maturation medium on the concentration of glucose, pyruvate, lactate and glutamine

After removing the COCs from maturation medium, media from all experimental treatments were collected and further measured for glucose, pyruvate, lactate and glutamine.

#### Experiment 3. Effects of LPS in maturation medium on in vitro development of oocyte

In this experiment, various concentrations (0, 0.01, 0.1, 1, and 10 μg/mL) of LPS were applied during oocyte maturation *in vitro* in order to define the dose of LPS with deleterious effect on the oocyte developmental competence. The number of replicates was six per treatment.

#### Experiment 4. Effects of LPS in culture medium on in vitro development of presumable zygotes

Following IVM and IVF, various concentrations (0, 0.01, 0.1, 1, and 10 μg/mL) of LPS were added to IVC medium. The rates of cleavage and development to the blastocyst stage were recorded at days 3 and 9 post insemination, respectively. Seven replicates were performed per treatment.

## Results

### Experiment 1. Effects of LPS in maturation medium on the nuclear status and GSH content of oocytes

The effect of LPS on the meiotic progression during oocyte maturation is presented in Table 1.

**Table 1:** Effect of LPS in maturation medium on proportion of oocytes reaching to the MII stage.

**Groups**	**No.**	**MII**	**Odd ratio**
	**Oocyte**	**n (%)**	
Control	241	184 (76.34)	-
0.01	203	158 (77.83)	1.09
0.1	218	154 (70.64)	0.75
1	212	146 (68.86)	0.69
10	193	128 (66.32)	0.61^*^

MII: metaphase-II.

* p-values different from the control with *P<0.05*.

Although there was no difference (*P≥0.05*) in the rate of meiotic progression to MII between the control, 0.01 and 0.1 μg/mL of LPS treatments, the value was lower when oocytes were matured in the medium supplemented with 1 (68.86%) and 10 (66.32%) μg/mL LPS when compared to the control group (76.34%). A significant difference (*P<0.05*) was observed between the control and treatment with 10 μg/mL of LPS.

**Figure 1:**
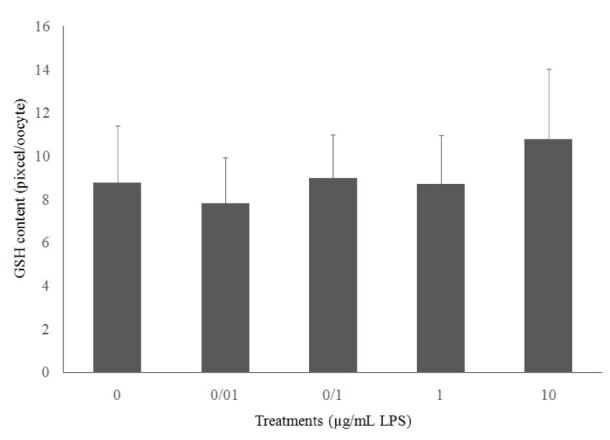
The effect of different LPS concentrations on the GSH level in oocytes after *in vitro* maturation.

The intracellular concentration of GSH measured after IVM is shown in Figure 1. There were no significant differences (*P≥0.05*) in the intracellular content of GSH between treatments.

### Experiment 2. Effects of LPS in maturation medium on the concentration of glucose, pyruvate, lactate and glutamine

The effects of LPS on the concentrations of glucose, pyruvate, lactate and glutamine are shown in Table 2. The concentration of the above-mentioned metabolites was not affected (*P≥0.05*) by LPS supplementation.

**Table 2:** Glucose, pyruvate, lactate and glutamin concentrations of maturation medium affected by various LPS levels.

**Metabolites**	**LPS treatment (µg/mL)**					**Standard**	**P-value**	**P-value**	**P-value**
**(pmol/mL)**	**0**	**0.01**	**0.1**	**1**	**10**	**Error**	**linear**	**quad**	**cub**
Glucose	83.33	82.66	80.66	80	80	0.96	0.21	0.74	0.77
Pyruvate	0.2	0.28	0.22	0.22	0.28	0.02	0.54	0.91	0.25
Lactate	49.93	50.76	50.6	51.3	51.46	4.12	0.89	0.37	0.98
Glutamin	549	533	522	513	518	14.68	0.48	0.74	0.93

No difference (*P≥0.05*) was observed among treated.

### Experiment 3. Effects of LPS in maturation medium on the in vitro development of oocyte

Our results demonstrate that different concentrations of LPS had no effect (*P≥0.05*) on the cleavage rate when compared to the control group. However, LPS addition significantly reduced the number of fertilized oocytes that reached the blastocyst stage. Treatments of maturation medium with 1 and 10 μg/mL LPS showed significant differences (*P<0.05*) in the blastocyst formation when compared with the control group (Table 3).

**Table 3:** Effect of LPS concentrations in *in vitro* maturation medium on proportion of cleavage and blastocyst rates of oocytes.

**Group**	**No.**	**Cleaved**	**Odd ratio**	**Blastocyst**	**Odd ratio**
	**Oocyte**	**n (%)**		**n (%)**	
Control	109	93 (85.32)	-	40 (36.69)	-
0.01	114	92 (80.70)	0.71	39 (34.21)	0.89
0.1	112	88 (78.57)	0.63	34 (30.35)	0.75
1	110	85 (77.27)	0.58	19 (17.27)^**^	0.36
10	114	86 (75.43)	0.52	16 (14.03)^***^	0.28

** p-values different from the control with *P<0.01*;

*** p-values different from the control with *P<0.001*.

### Experiment 4. Effects of LPS in culture medium on in vitro development of presumable zygotes

Effects of LPS in culture medium on cleavage rate and progression of embryos to the blastocyst stage are presented in Table 4. The increasing dose of LPS in the culture medium did not show any detrimental effect (*P≥0.05*) on the proportion of cleaved oocytes as well as the proportion of oocytes in blastocyst stage as observed in experiment 3.

**Table 4:** Effect of LPS concentrations in *in vitro* culture medium on proportion of cleavage and blastocyst rates of oocytes.

**Group**	**No.**	**Cleaved**	**Odd ratio**	**Blastocyst**	**Odd ratio**
	**Oocyte**	**n (%)**		**n (%)**	
Control	79	61 (77.21)	-	34 (43.03)	-
0.01	73	60 (82.19)	1.36	32 (43.83)	1.033
0.1	76	65 (85.52)	1.74	37 (48.68)	1.25
1	77	61 (79.22)	1.12	30 (38.96)	0.84
10	76	63 (82.89)	1.43	27 (35.52)	0.72

No difference (*P≥0.05*) was observed among treated.

## Discussion

The present study examined the effect of adding LPS during oocyte maturation on the oocyte developmental competence. LPS at higher concentrations (i.e. 1 and 10 µg/mL) reduced the competence of oocytes to the blastocyst stage. This finding is in agreement with Magata and Shimizu’s (2017) study, in which the proportion of oocytes reaching the MII stage decreased in response to the increased levels of LPS. Similarly, our lower rates of blastocyst formation in response to higher LPS level complement the findings of the aforementioned study (Magata and Shimizu, 2017). Injection of 10 μg/mL LPS into bovine oocytes showed that LPS reduced the development ability of oocyte (Roth et al., 2015). Although 1 µg/mL LPS had no suppressive effects on the cleavage rate, this concentration significantly reduced the developmental competence of zygotes to morula or blastocyst stages. Our results revealed that 1 µg/mL LPS can not only reduce the rate of blastocyst formation but also decrease the quality of blastocysts (Mokhtari et al., 2020). Using LPS on the day of zygote formation caused a reduction in blastocyst cell numbers (Williams et al., 2011). Although *in vitro* exposure to LPS did not affect the early embryo development but *in vivo* LPS reduced the cleavage rate. Findings of this study showed that treatment with 1 and 5 μg/mL LPS during IVC did not affect the embryonic development similarly to Rincon et al. (2019). Moreover, LPS can stimulate oxidative stress by activating the *NF-κB* and *MAPK* signaling pathways (Gehart et al., 2010; Kastl et al., 2014). Therefore, LPS can induce apoptosis by increasing the expression of oxidative stress genes and sensitive proteins (Raza et al., 2016). Several studies have demonstrated the positive effects of high GSH levels on embryogenesis through protecting the cells against oxidative stress (Gasparrini et al., 2006). Synthesis of GSH during oocyte maturation has been reported in porcine, bovine, ovine and caprine oocytes (Luberda, 2005). In mammals, oxidative stress severely interferes with the gamete viability and embryo development. A group of antioxidant enzymes and non-enzymatic molecules protects gametes and embryos against reactive oxygen species (ROS) inflicting potential damage during oocyte maturation and early stage of embryogenesis (Guerin et al., 2001). In this experiment, the increased concentration of GSH in response to a high level of LPS could be a mechanism to confront the inflammatory response created in this experiment. Although, we did not measure the ROS concentration in the current study, lower GSH concentrations of oocytes subjected to treatment with lower LPS concentrations (i.e. 0.01 and 0.1 µg/mL) might be related to the fact that GSH was used to scavenge ROS.

The surrounding environment of COCs during maturation either *in vivo* or *in vitro* may have profound effects on the success of fertilization and subsequent embryo development (Sutton-McDowall et al., 2010). The rapid and dynamic nature of the final stages of oocyte maturation comes along with the need for metabolites such as fatty acids, amino acids, electrolytes, purines and pyrimidines. Glucose, in particular is an important energy substrate and its addition to the medium in appropriate concentrations leads to improved maturation and blastocyst development (Khurana and Niemann, 2000; Zheng et al., 2001). Amino acids may be utilized by oocytes as an energy source for the cumulus cells, and play an important role in the amino acid flux into the oocyte (Colonna and Mangia, 1983). GSH is a thiol tripeptide comprising cysteine, proline and glutamine, and is an important reducing agent and scavenger that protects cells against ROS and is necessary for the expansion of the cumulus cells. GSH synthesis is highly dependent on the levels of cysteine, a highly unstable amino acid that is readily oxidized to cystine. In our study, none of the analyzed metabolites were affected by LPS treatments which demonstrates that the LPS effect on the oocyte developmental competence may not be mediated through energy pathways and energy substrate availability. The other possible explanation can be the related to the fact that measuring these metabolites is not sensitive enough to show any possible molecular changes behind. Therefore, evaluating the expression of genes involved in the pathways would further clarify the involved mechanism in more detail. It has been reported that bovine granulosa cells express the *TLR4* receptor complex and respond to LPS through phosphorylation of the *TLR* signaling components *p38*, extracellular signal-regulated kinase and increase the *IL-6* and *IL8* transcripts (Bromfield and Sheldon, 2011). LPS was reported to affect the intracellular redox status and increase apoptosis through enhancing pro-apoptotic factors (Raza et al., 2016). The concentration of inflammatory cytokines was not measured in the current study while it can be hypothesized that the deleterious effects of LPS on the oocyte developmental competence is likely to be mediated via inflammatory pathways. Low doses of LPS might exhibit beneficial effects through triggering antioxidant processes. In this study as well as in the report by Magata and Shimizu (2017) the concentrations of LPS were in a range similar to cows with acute endometritis (Sheldon et al., 2009) which may be not low enough to just activate the antioxidant system. Hence in order to observe any possible beneficial effects of LPS on the oocyte developmental competence, lower concentrations of LPS should be used in future studies.

## Conclusion

In summary, the data presented here have shown that LPS exhibits detrimental effects on the maturation ability and developmental competence of oocytes in a dose dependent manner. Our findings show that such deleterious effects of LPS are probably not mediated through the energy related pathways.
